# Dual-energy CT of acute bowel ischemia—influence on diagnostic accuracy and reader confidence

**DOI:** 10.1007/s00330-024-11217-1

**Published:** 2024-11-27

**Authors:** Moritz Oberparleiter, Jan Vosshenrich, Hanns-Christian Breit, Philippe Kaiser, Paul Hehenkamp, Dorothee Harder, Daniel T. Boll, Christoph J. Zech, Markus M. Obmann

**Affiliations:** Department of Radiology and Nuclear Medicine, University Hospital Basel, University of Basel, Basel, Switzerland

**Keywords:** Tomography (X-ray computed), Mesenteric ischemia, Intestines

## Abstract

**Objectives:**

This study evaluates the advantages in diagnostic accuracy, confidence, and reading time of additional dual-energy CT-derived reconstructions for assessing acute bowel ischemia.

**Methods and materials:**

This retrospective study includes 25 patients with surgically proven acute bowel ischemia and 25 gender- and age-matched controls who underwent biphasic abdominal dual-energy CT. Two fellowship-trained abdominal radiologists and two residents evaluated all cases with and without additional dual-energy CT-derived iodine maps and virtual non-contrast images. Diagnostic confidence was rated on a 10-point Likert scale. Reading time was recorded. The inter-reader agreement was assessed using Fleiss’ kappa. Sensitivity and specificity were compared using McNemar’s test, reader confidence, and reading times with the Wilcoxon signed-rank test.

**Results:**

For conventional images alone, the inter-reader agreement was moderate (*κ* = 0.58), with a sensitivity of 77% (95% CI: 67.5–84.8%) and specificity of 90% (95% CI: 82.4–95.1%). Adding dual-energy CT images, inter-reader agreement increased to substantial (*κ* = 0.69), sensitivity increased significantly to 89% (95% CI: 81.2–94.4%, *p* = 0.02), while specificity increased non-significantly to 93% (95% CI: 86.1–97.1%, *p* = 0.51). Diagnostic confidence increased significantly from 8 (IQR: 6–8) to 9 (IQR: 8–9) (*p* < 0.01). Equivalent diagnostic accuracy and confidence increases were observed for fellowship-trained and resident radiologists. A non-significant increase in mean reading time per case from 196 s to 201 s was observed (*p* = 0.30).

**Conclusion:**

Additional dual-energy CT reconstructions increase diagnostic accuracy and confidence without increasing reading time when evaluating suspected acute bowel ischemia. Both experienced and resident readers benefit from dual-energy CT images.

**Key Points:**

***Question*** *There are too few clinical studies assessing the diagnostic accuracy of dual-energy CT (DECT) to recommend its use for evaluating suspected acute bowel ischemia*.

***Findings*** *Adding DECT-derived iodine maps and virtual-non-contrast images increase reader sensitivity and confidence while maintaining high specificity when evaluating for acute mesenteric ischemia*.

***Clinical relevance*** *Dual-energy CT should be used to investigate suspected acute bowel ischemia. Both diagnostic accuracy and confidence can be increased independent of reader experience without significantly increasing reading time*.

## Introduction

Acute bowel ischemia (ABI) is a rare condition, accounting for 0.1–0.2% of acute surgical admissions [1]. However, it is associated with high mortality (approximately 50%) and morbidity [1]. Clinical diagnosis remains challenging due to the absence of specific clinical findings or plasma biomarkers [2, 3]. The etiology of ABI can be broadly categorized into occlusive and nonocclusive ABI. Causes for occlusive ABI include arterial emboli, arterial or venous thrombosis, and torsion or compression of mesenteric vessels. In cases of nonocclusive ABI, a vascular occlusion cannot be visualized. It is presumed to be due to vasoconstriction in combination with a low-flow state, often seen in critically ill patients. Hence, radiological examinations are critical for diagnosis, with CT being the modality of choice for detecting ABI [4]. Accurate and rapid diagnosis facilitated by CT allows for early therapeutic intervention and improves overall survival [5, 6]. Standard practice involves acquiring scans in arterial and portal venous phases when ABI is suspected clinically [7, 8]. Some authors recommend using additional non-contrast scans to detect intramural hemorrhage [9]. Nevertheless, ABI may present with subtle imaging findings, complicating confident CT diagnosis of ABI [10].

Data of two different beam energies is acquired in dual-energy computed tomography (DECT) imaging. By leveraging the differential attenuation at the two photon energies, tissue composition can be further characterized. Using material decomposition, iodine uptake can both be visualized and quantified with iodine maps, and at the same time, virtual non-contrast images (VNC) can be generated [11]. These techniques enhance the visualization of critical findings indicative of ABI, such as intramural hemorrhage or only subtle reduced bowel wall enhancement [12]. Several publications suggest the use of DECT for diagnosing ABI. However, these are primarily case reports, case series, expert opinions, or theoretical considerations [12–14]. There is a lack of clinical studies assessing the diagnostic accuracy of DECT, with currently conflicting results [15, 16]. This lack explains why current guidelines have not yet included a recommendation for using DECT in ABI.

The purpose of this study was to compare the diagnostic performance of conventional CT images vs the additional use of DECT image reconstructions. Additionally, diagnostic accuracy, reader confidence, and reading times between the two image sets will be assessed both by experienced readers and radiologists in training.

## Materials and methods

This retrospective, single-center study was approved by the institutional review board, and the need for informed consent was waived.

### Study population

**Fig. 1:**
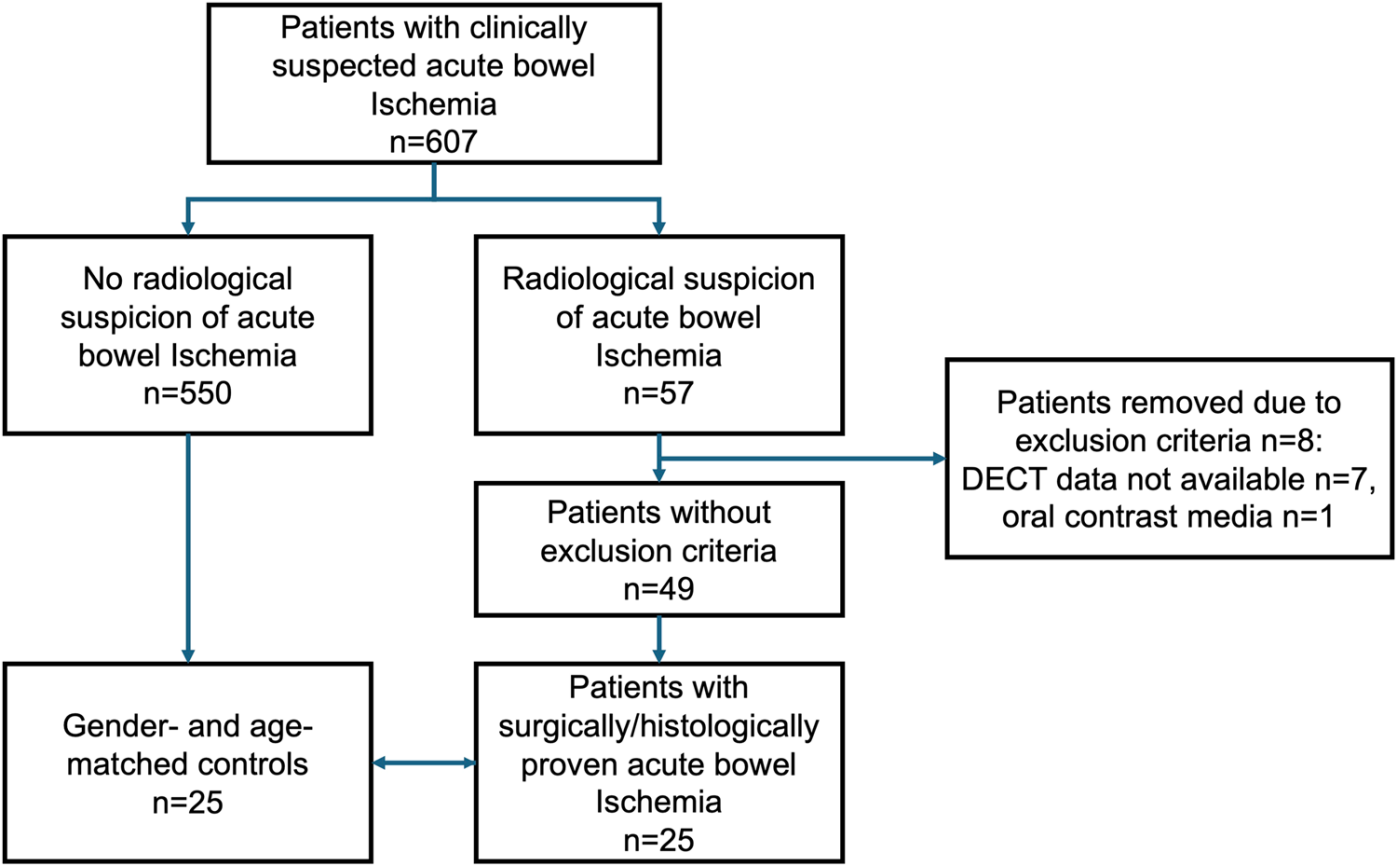
Flowchart of the recruitment of the study population

Searching our picture archiving and communication system, all patients with clinical suspicion of ABI who underwent biphasic (arterial and portal venous phase) abdominal CT between September 2015 and March 2023 were identified and primarily included in this study (Fig. 1). The radiological reports of these studies were evaluated for the radiological suspicion of ABI (*n* = 57) or no radiological suspicion of ABI (*n* = 550) and divided into two groups accordingly. Patients meeting one or more of the following criteria were excluded from the study: (a) patients younger than 18 years, (b) documented refusal of the patient, (c) oral contrast agent administered during the exam, or (d) incomplete imaging data. After applying the exclusion criteria, 49 studies with suspicion of ABI remained and were correlated with surgical and pathological reports. Ultimately, 25 patients with available DECT data and surgically or histologically proven acute mesenteric ischemia were included. For each patient with proven ABI, a matched control was selected from the cohort of patients without ABI (*n* = 550). Controls were matched based on gender and age, with the control being the patient of the same gender and closest in age at the time of CT imaging. Laboratory information acquired immediately prior to the CT examination for all 50 patients was retrieved from the local hospital information system, including white blood cell count (WBC, 10^6^/L), serum lactate (mmol/L), and C-reactive protein (CRP, mmol/L).

### CT image acquisition

DECT was performed on four different scanners with two different types of DECT platforms in supine position: (a) dual-source 192-MDCT scanner (SOMATOM Force; Siemens Healthineers) with 37 CT scans, (b) dual-source 128-MDCT scanner (SOMATOM Definition Flash; Siemens Healthineers) with 1 CT scan, (c) split-filter 128-MDCT scanners (SOMATOM Definition AS + /Edge, Siemens Healthineers) with 12 CT scans.

Both arterial and portal venous phases were acquired as part of our standardized imaging protocol for ABI (Table 1). Single-energy CT with tube voltages ranging from 70 kVp to 100 kVp was utilized for the arterial phases. For the portal venous phases, DECT was used with tube voltages set at 100 and 140 (flash)/150 (force) kVp for dual-source DECT and 120 kVp for split-filter DECT. In all instances, an automatic exposure control system regulated the tube current. All patients received intravenous iodinated contrast medium and no oral contrast medium. Based on the patient’s body weight (1.1–1.3 mL contrast medium/kg body weight), a volume of 70–110 mL of either Iopamidol (Iopamiro370, Bayer) or Iopromide (Ultravist370, Bayer), each containing 370 mg of iodine per milliliter, was administered at a flow rate of 3–4 mL/s (see Supplementary Table 1). After a trigger threshold of 100 HU was reached in the aorta at the height of the diaphragm, the arterial phase was acquired with a delay of 9 s. The portal venous phase was acquired with a fixed time delay of 55 s after reaching the threshold in the aorta.

**Table 1:** CT acquisition parameters and radiation dose

	Dual-source 192-MDCT	Dual-source 128-MDCT^1^	Split filter 128-MDCT
Contrast			
Contrast volume, (mL)	90.8 ± 12.8	70	75.4 ± 9.88
Flow rate, (mL/s)	3.85 ± 0.33	4	3.79 ± 0.26
Overall			
CTDIvol, (mGy)	18.1 ± 9.14	13.1	13.2 ± 2.76
DLP, (mGy·cm)	951 ± 545	563.5	674 ± 191
Arterial phase			
kVp	70–100	100	100
Ref. mAs	223 ± 82.5	158	172 ± 20.7
mAs	244 ± 72.2	85	155 ± 72.2
CTIvol, (mGy)	6.34 ± 3.68	3.50	5.19 ± 1.81
DLP, (mGy·cm)	316 ± 191	166	251 ± 103
Portal venous phase			
kVp	100/Sn150	100/Sn140	AuSn120
Ref. mAs	177 ± 14.2/88.7 ± 7.08	180/153	420
mAs	195 ± 93.9/88.9 ± 38.7	113/101	392 ± 5.82
CTDivol, (mGy)	11.8 ± 5.60	9.61	8.01 ± 0.966
DLP, (mGy·cm)	583 ± 297	564	386 ± 70.3

Values are expressed as means ± standard deviation

*CTDI*_*vol*_ CT dose index, *DLP* dose length product, *Sn* additional tin-filtration, *AuSn* split-filter out of gold and tin placed in front of the tube output

^1^ Only one patient was scanned using the dual-source 128-MDCT

Arterial phase images were reconstructed with a slice thickness of 1.5 mm and an interval of 1 mm using a medium soft-tissue kernel (Br36). Conventional images in the portal venous phase were reconstructed as 120 kVp-equivalent images as a mix of the high- and low-energy datasets with a slice thickness of 5 mm and an interval of 2.5 mm using the same soft-tissue kernel (Br36). VNC images and iodine maps were automatically reconstructed from the high- and low-energy dataset using dedicated software (Syngo.via, version 40 A, Siemens Healthcare) with a quantitative soft-tissue kernel (Br36), a slice thickness of 5 mm, and an interval of 2.5 mm. The matrix size was 512 × 512 in all cases, and only axial reconstructions were provided.

### CT image interpretation

Four independent readers conducted the imaging evaluation. Two senior readers were fellowship-trained abdominal radiologists with 7 years and 8 years of experience. The other two readers were residents in their second and third years of training. All reviewers were blinded to any clinical information. A secondary imaging platform (NORA) was used for reading [17]. This platform allowed automatic recording of reading times and convenient evaluation of imaging features through a questionnaire on the same screen as the images.

Scans of patients with and without ABI were anonymized and presented in a randomized sequence for evaluation in two reading sessions. In the first session, readers assessed only the conventional imaging data. In the second session, following a washout period of at least seven days, the readers assessed the imaging data, including the additional DECT data, featuring VNC images and iodine maps. Readers were required to identify the presence or absence of ABI in both sessions. Diagnostic confidence was reported using a 10-point Likert scale, where one represented the lowest, and ten represented the highest confidence. Reading times were automatically recorded.

Additionally, the readers were instructed to evaluate the presence of the following radiological features of bowel ischemia in each scan: celiac trunk occlusion, superior mesenteric artery (SMA) occlusion, inferior mesenteric artery (IMA) occlusion, small bowel obstruction, large bowel obstruction, small bowel distension (> 3 cm), large bowel distension (> 6 cm, cecum/sigmoid > 9 cm), reduced mural enhancement in the portal venous phase, intestinal pneumatosis, porto-mesenteric gas, bowel wall thinning, bowel wall thickening, intramural hemorrhage, mesenteric congestion, and mesenteric fluid.

### Reference standard

For ABI, the reference standards were surgery, pathology, and autopsy reports retrieved from the electronic patient records. The mention of ABI in one of the reports was considered the gold standard for diagnosis. For the control group, such reports were not always available. In these cases, the reference standard was CT when the radiological findings were unequivocal or the final clinical diagnosis in the discharge summaries.

### Statistical analysis

Continuous data are presented as means with standard deviation, and discrete data as medians with interquartile ranges. Inter-reader agreement for detecting bowel ischemia was assessed using Fleiss’ kappa. A kappa value of 0.41–0.60 was considered moderate, while a kappa value of 0.61–0.80 was considered substantial [18]. Sensitivity, specificity, and accuracy were reported as percentages with 95% confidence intervals (CI). Sensitivity and specificity were compared using McNemar’s test, and accuracy was evaluated with a *Z*-test. Reading time was reported in seconds with a standard deviation. The differences in reading times between conventional images alone and additional DECT images, as well as differences in reader confidence, were compared using the Wilcoxon signed-rank test. *p*-values < 0.05 were considered statistically significant. Adjustments for multiple testing using Bonferroni correction were applied for individual reader analyses. Statistical analyses were performed using SPSS Statistics software (version 29, IBM).

## Results

After applying exclusion criteria, the final cohort of this study comprised twenty-five patients with surgically or pathologically proven ABI and twenty-five gender- and age-matched controls without ABI (Fig. 1). A total of 26 women and 24 men were included. Table 2 provides an overview of the demographics and clinical parameters of the patient and control cohorts. The study population had a mean age of 66 ± 15 years. No significant differences in age or gender were observed between the groups. Patients with ABI and controls demonstrated significant differences in CRP (*p* = 0.01) and serum lactate (*p* = 0.01), whereas differences in WBC (*p* = 0.16) were not statistically significant. Vascular occlusion was present in 56% of patients with ABI, while the remaining cases were due to non-occlusive mesenteric ischemia (NOMI). The final diagnoses of the control group included 5 cases of small bowel obstruction, 3 cases of paralytic ileus, 3 cases of gastroenteritis, 3 cases of constipation/coprostasis, and 1 case each of large bowel obstruction, cholangitis, urinary retention, sepsis, gastritis, pancreatitis, decompensated liver cirrhosis and renal bleeding. In 3 cases the final diagnosis remained unclear, but the patient did not develop any mesenteric ischemia in clinical follow-up. The mean radiation dose of the CT acquisition (total DLP and CTDIvol) was 834 ± 421 mGy·cm and 16.2 ± 7.52 mGy.

**Table 2:** Characteristics of the 25 patients with proven ABI and the 25 control subjects

	Overall, (*n* = 50)	Ischemia, (*n* = 25)	Controls, (*n* = 25)
Age (years, range)	66 ± 15	66 ± 15	66 ± 15
Female	26 (52)	13 (52)	13 (52)
Male	24 (48)	12 (48)	12 (48)
Inpatients/emergency admissions	24 (48)/26 (52)	17 (68)/8 (32)	7 (28)/18 (72)
Laboratory data			
WBC, (10^9^/L)	12.3 ± 6.7	14.4 ± 8.5	10.1 ± 3.0
CRP, (mg/mL)	73.1 ± 102.4	100.0 ± 110.8	46.3 ± 87.4
Lactate, (mmol/L)	2.91 ± 2.52	3.83 ± 3.01	1.99 ± 1.46
Treatment/outcome			
Surgery	–	23 (92)	–
Conservative/palliative	–	2 (8)	–
Death	14 (28)	12 (48)	2 (8)
Proof of ischemia			
Operative report	–	21	–
Pathology report	–	6^1^	–
Autopsy	–	2	–
Location of ischemia			
Small bowl	–	18^2^ (64)	–
Large bowel	–	9^2^ (28)	–
Etiology of ischemia			
Occlusive	–	14 (56)	–
Non-occlusive	–	11 (44)	–

Numbers in parentheses represent percentages. Values are expressed as means ± standard deviation

*WBC* white blood cell count, *CRP* C-reactive protein

^1^ Four of the patients with histologically proven ABI also had operative reports that confirmed ABI

^2^ Two patients showed small and large bowel ischemia

### Inter-reader agreement

The overall inter-reader agreement for the diagnosis of ABI was moderate (*κ* = 0.58). The inter-reader agreement was also moderate when using only the conventional imaging data (*κ* = 0.46). However, the inter-reader agreement improved to substantial (*κ* = 0.69) when the additional DECT data was utilized. The fellowship-trained abdominal radiologists demonstrated substantial inter-reader agreement (*κ* = 0.71), while the residents achieved only moderate inter-reader agreement (*κ* = 0.58).

### Diagnostic performance for ABI

Overall sensitivity and specificity for diagnosing acute mesenteric ischemia for all readers were 77.0% (95% CI: 68.8–85.2%) and 90.0% (95% CI: 84.1–95.9%), respectively, using conventional images alone (Table 3). With the inclusion of additional DECT data, sensitivity increased to 89.0% (95% CI: 82.9–95.1%) (*p* = 0.02), while specificity showed a non-significant increase to 93.0% (95% CI: 88.0–98.0%) (*p* = 0.51). Accuracy increased from 83.5% to 91.0% (*p* = 0.02) using additional DECT data.

**Table 3:** Diagnostic performance of conventional and additional dual-energy CT for the assessment of ABI

	Conventional	Additional DECT	*p*-values
	Value (95% CI)	Value (95% CI)	
Senior radiologists			
Sensitivity	78.0 (64.0–88.5)	94.0 (83.5–98.8)	0.02
Specificity	100.0 (92.9–100.0)	100.0 (92.9–100.0)	1
Accuracy	89.0 (81.2–94.4)	97.0 (91.5–99.4)	0.03
Residents			
Sensitivity	76.0 (61.8–86.9)	84.0 (70.9–92.8)	0.42
Specificity	80.0 (66.3–90.0)	86.0 (73.3–94.2)	0.51
Accuracy	78.0 (68.6–85.7)	85.0 (76.5–91.4)	0.20
All readers			
Sensitivity	77.0 (67.5–84.8)	89.0 (81.2–94.4)	0.02
Specificity	90.0 (82.4–95.1)	93.0 (86.1–97.1)	0.51
Accuracy	83.5 (77.6–88.4)	91.0 (86.2–94.6)	0.02

All values are presented as percentages

*PPV* positive predictive value, *NPV* negative predictive value

When considering only the fellowship-trained abdominal radiologists, the sensitivity increased from 78.0% (95% CI: 64.0–88.5%) to 94.0% (95% CI: 83.5–98.8%) (*p* = 0.02). While the sensitivity for residents also increased from 76.0% (95% CI: 61.8–86.9%) to 84.0% (95% CI: 70.9–92.8%), the increase did not reach statistical significance (*p* = 0.42). The specificity for the fellowship-trained radiologists remained at 100% (95% CI: 92.9–100.0%) for both conventional and additional DECT data. For the residents, specificity increased from 80.0% (95% CI: 66.3–90.0%) to 86.0% (95% CI: 73.3–94.2%); this change was also not statistically significant (*p* = 0.51).

### Radiological signs of ABI

Inter-reader agreements for individual imaging features of ABI are presented in Table 4. For most individual signs, only weak inter-reader agreement was observed. However, inter-reader agreement was substantial for celiac trunk occlusion and moderate for SMA occlusion, porto-mesenteric gas, and intestinal pneumatosis.

**Table 4:** Comparison of the inter-reader agreement between conventional data and additional DECT data

	Overall, (*n* = 100)					Conventional, (*n* = 50)	Additional DECT, (*n* = 50)
	Reader 1	Reader 2	Reader 3	Reader 4	kappa (95% CI)	kappa (95% CI)	kappa (95% CI)
Arterial vessel occlusion							
Celiac trunk occlusion	5	6	7	6	0.82 (0.74–0.903)	0.82 (0.71–0.94)	0.82 (0.71–0.94)
SMA occlusion	5	8	12	7	0.64 (0.56–0.718)	0.64 (0.53–0.75)	0.64 (0.53–0.75)
IMA occlusion	4	4	18	16	0.38 (0.30–0.46)	0.35 (0.24–0.47)	0.41 (0.29–0.52)
Bowel imaging features							
Small bowel obstruction	29	32	53	41	0.54 (0.46–0.62)	0.50 (0.39–0.61)	0.58 (0.47–0.69)
Large bowel obstruction	4	3	12	2	0.31 (0.23–0.39)	0.21 (0.09–0.32)	0.48 (0.37–0.59)
Small bowel distension	46	44	56	47	0.59 (0.52–0.68)	0.59 (0.48–0.71)	0.59 (0.48–0.71)
Large bowel distension	6	12	13	9	0.50 (0.42–0.58)	0.48 (0.37–0.60)	0.52 (0.41–0.63)
Intestinal pneumatosis	16	20	13	9	0.69 (0.61–0.77)	0.65 (0.53–0.76)	0.73 (0.61–0.84)
Bowel wall thinning	25	20	15	10	0.19 (0.11–0.27)	0.16 (0.04–0.27)	0.23 (0.12–0.34)
Bowel wall thickening	33	47	34	30	0.32 (0.24–0.40)	0.26 (0.14–0.37)	0.38 (0.27–0.50)
Reduced portal venous mural enhancement	32	47	45	20	0.39 (0.31–0.47)	0.33 (0.22–0.44)	0.44 (0.33–0.55)
Intramural hemorrhage	1	23	11	13	0.05 (−0.03 to 0.13)	0.03 (−0.09 to 0.14)	0.07 (−0.04 to 0.19)
Veins and mesentery							
Portomesenteric gas	9	15	11	8	0.73 (0.65–0.81)	0.70 (0.59–0.82)	0.75 (0.64–0.87)
Mesenteric congestion	14	53	41	39	0.39 (0.31–0.47)	0.29 (0.17–0.40)	0.49 (0.37–0.60)
Mesenteric fluid	24	58	45	57	0.52 (0.44–0.60)	0.53 (0.42–0.65)	0.50 (0.38–0.61)

Absolute counts of findings are given and equivalent to percentages, as the number of total assessed studies was *n* = 100

*SMA* superior mesenteric artery, *IMA* inferior mesenteric artery

### Diagnostic confidence

**Fig. 2:**
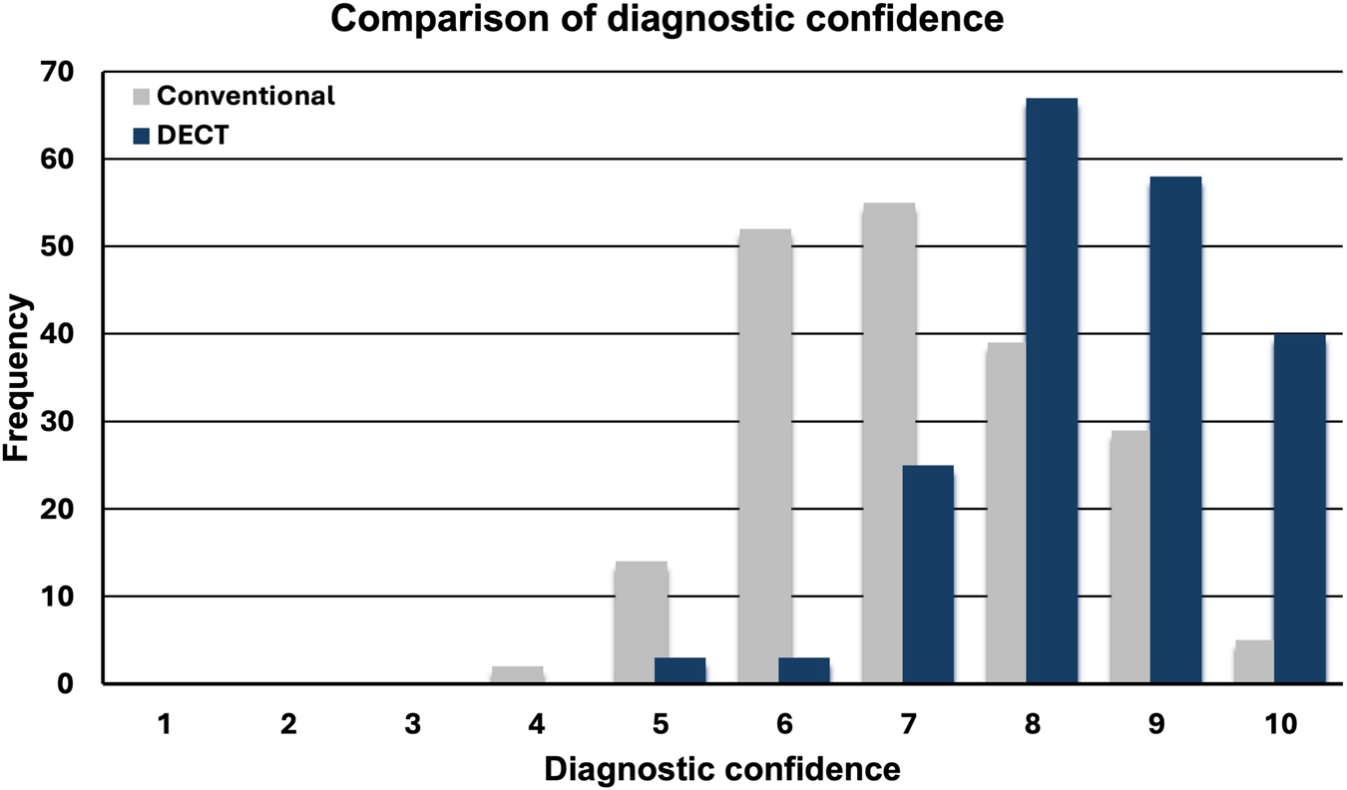
Histogram-plot of the distribution of readers’ diagnostic confidence of cases with conventional CT and additional DECT. Diagnostic confidence is graded on a 10-point Likert scale

Overall diagnostic confidence for the diagnosis of ABI increased from 8 (IQR, 6–8) using conventional data to 9 (IQR, 8–9) with the addition of DECT data (*p* < 0.001); see also Fig. 2. The diagnostic confidence for the fellowship-trained abdominal radiologists was 8 (IQR, 7–9) with conventional data and increased to 9 (IQR, 8–10) with the addition of DECT data (*p* < 0.001). The residents reported a lower diagnostic confidence of 6 (IQR, 6–7) with conventional data, which increased to 8 (IQR, 7–8.75) with additional DECT data (*p* < 0.001). After correction for multiple testing, the diagnostic confidence for each individual reader still increased significantly (*p* < 0.001) when using additional DECT images: for Reader A from 7 (IQR, 7–8) to 9 (IQR, 8–9), for reader B from 9 (IQR, 8–9) to 10 (IQR, 9–10), for reader C from 6 (IQR, 6–7) to 8 (IQR, 7–9), and for reader D from 6 (IQR, 5–7) to 8 (IQR, 7–8).

### Reading time

**Fig. 3:**
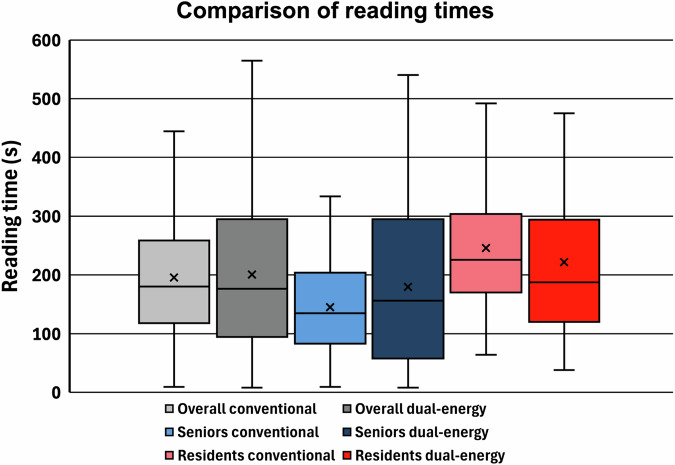
Comparison of reading times of conventional vs additional DECT data

The mean overall reading time per case increased slightly from 196 s to 201 s with additional DECT images (*p* = 0.30). Notably, the average reading times did not increase for all readers (Fig. 3). In fact, for reader A (senior radiologist), reading time decreased from 107 ± 76 s to 81 ± 77 s; similarly, for reader D (resident), it decreased from 223 ± 85 s to 153 ± 68 s. In contrast, the mean reading times were increased for reader B (senior radiologist) from 182 ± 57 s to 276 ± 107 s and for reader C (resident) from 268 ± 139 s to 291 ± 154 s.

## Discussion

In our study, we compared the diagnostic performance of conventional biphasic CT vs using additional DECT images to diagnose ABI. We found significantly higher diagnostic accuracy when using DECT images than conventional CT images alone (83.5% vs 91.0%, *p* = 0.02). A significant increase in sensitivity was observed when adding DECT images (77.0% vs 89.0%, *p* = 0.02), with specificity showing a non-significant increase (90.0% vs 93.0%, *p* = 0.51). Accordingly, inter-reader agreement increased from moderate when using conventional CT images (*κ* = 0.58) to substantial when adding DECT images (*κ* = 0.69). Including DECT images did not result in any significant increase in reading time (*p* = 0.30).

**Fig. 4:**
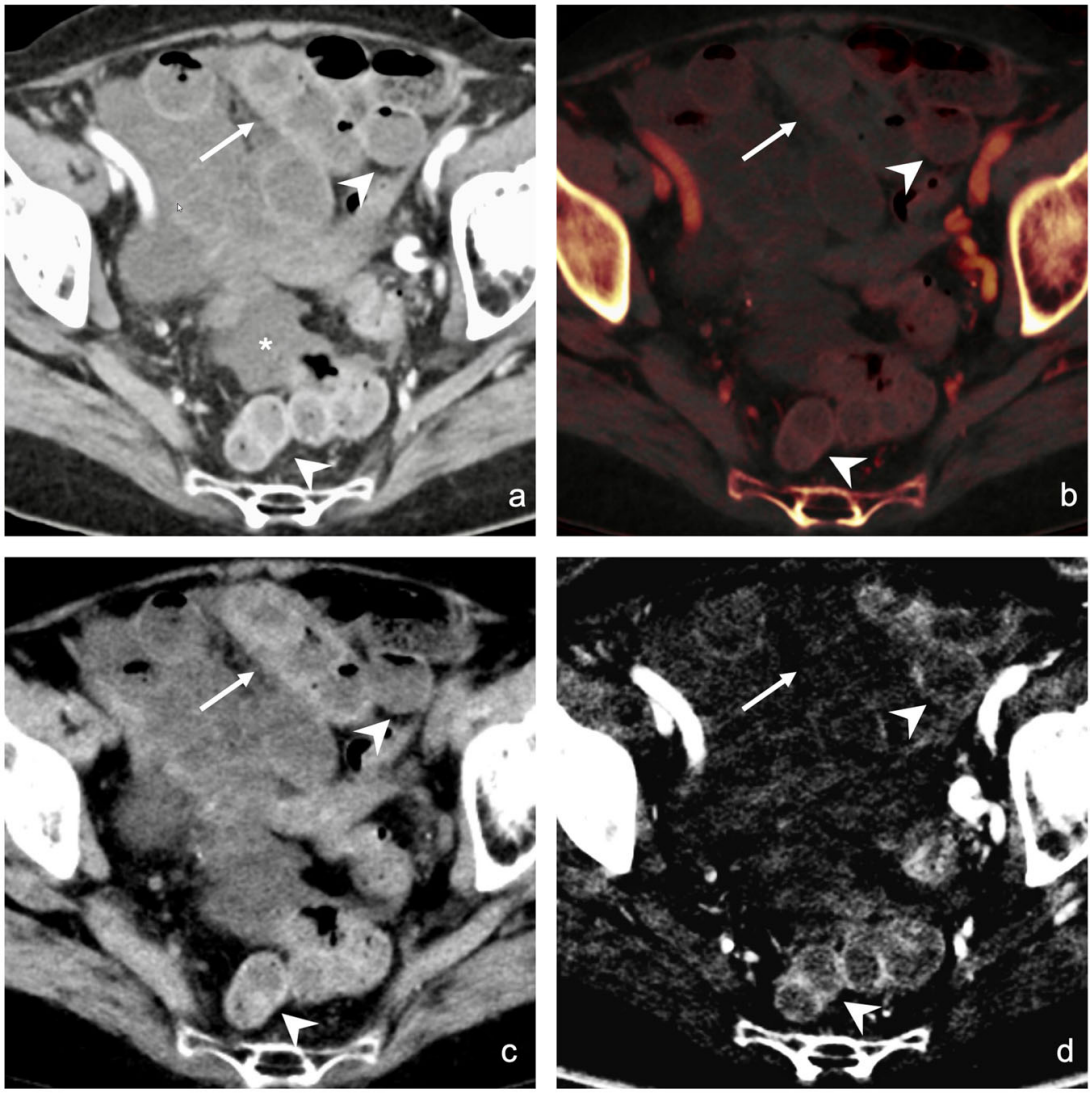
CT scan of a patient with ABI due to closed-loop small bowel obstruction. Arrow: ischemic bowel loop, arrowheads: non-ischemic bowel loops, asterisk: free peritoneal fluid. **a** Conventional CT image in the portal venous phase. Non-ischemic bowel loops and the ischemic segment exhibit similar attenuations and cannot be distinguished. **b** Color-coded iodine overlay image on top of the VNC image shows subtle hypoenhancement of the ischemic bowel loop; however, the density in the VNC image is hard to differentiate from the iodine signal due to enhancement. **c** VNC image: the ischemic bowel loop (arrow) demonstrates high attenuation on the VNC images. Non-ischemic bowel loops (arrowheads) demonstrate normal residual soft tissue density on the VNC image. **d** The iodine map shows no contrast media uptake in the ischemic bowel loop. Non-ischemic bowel loops (arrowheads) show normal iodine uptake. Thus, the mutual evaluation of VNC and iodine maps strongly suggests bowel ischemia with intramural hemorrhage, which is not readily appreciable on overlay images

Two prior studies assessed the diagnostic performance of conventional CT vs additional DECT, with conflicting outcomes for DECT image evaluation [15, 16]. Concordant with our results, Lourenco et al found improved diagnostic accuracy, confidence, and inter-reader agreement when adding DECT images. While increases in this previous study were not statistically significant, we could show statistically significant increases in overall sensitivity and accuracy. Our study included more than twice the number of patients with proven bowel ischemia. Furthermore, we included readers of different levels of training and assessed reading time, providing even stronger evidence for using DECT in ABI. The study by Lourenco used iodine maps and 40-keV virtual monoenergetic images (VMIs) but did not include VNC images [15]. However, VNCs may prove vital when assessing the bowel wall to identify iodine densities as artificial in the iodine map, especially in cases of intramural hemorrhage [12]; see also Fig. 4. The improved detection of intramural hemorrhages might explain the better diagnostic performance in our study. We omitted low-keV VMIs in our study as we do not use them routinely at our institution. While low-keV VMIs increase the iodine-based contrast between ischemic and non-ischemic bowel loops, this is even more pronounced in iodine images [19]. We therefore did not expect an additional increase in diagnostic accuracy. Furthermore, as is known from evaluations for other solid abdominal organs, the optimal keV level varies depending on the organ, pathology, and dual-energy CT scanner platform. Finding the optimal keV level for bowel ischemia would require additional analyses and was beyond the scope of this paper.

**Fig. 5:**
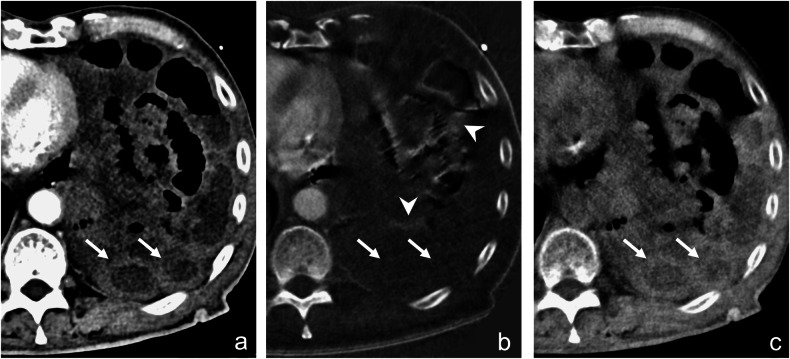
CT scan of a patient with ABI due to hiatal herniation. Arrows: ischemic bowel loops, arrowheads: artifacts mimicking iodine uptake at bowel wall-air interfaces. **a** Conventional CT image in the portal venous phase. The ischemic bowel loops (arrows) exhibit wall thickening without other clear signs of intestinal ischemia. Using only the conventional CT image, ischemia cannot be confirmed. **b** Iodine map reveals a lack of mural contrast media uptake in the fluid-filled ischemic bowel loops (arrows), confirming ischemia. Some bowel loops and adjacent areas show artificial iodine uptake due to artifacts arising at the bowel wall-air interfaces (arrowheads). Diagnostic performance might be reduced for readers lacking prior experience or training in DECT imaging, as such artifacts may be mistaken as true bowel wall enhancement. **c** VNC image: the ischemic bowel loops (arrows) demonstrate moderate attenuation on the VNC images, slightly higher than muscle, representing the underlying soft tissue density of the bowel wall with possible intramural hemorrhage

The more recent study by Xu et al reported conflicting findings to our study and the one from Lourenco et al, as they found no increase in diagnostic performance when adding DECT data [16]. Several reasons might explain this apparent contradiction. Xu et al included a small patient cohort with only 14 patients with confirmed bowel ischemia in their study. They used color-coded iodine maps overlaid on VNC images, while we provided iodine maps in grayscale and VNC images separately. Depending on the reader’s experience, it may be harder to differentiate subtle changes in the overlaid color-coded iodine maps from residual density on the VNC images, as is seen in cases with intramural hemorrhage. These shortcomings are aggravated by the fact that readers in their study were not explicitly trained on the pitfalls of DECT imaging, see Fig. 5. The scanner used by Xu et al was a rapid-kV-switching DECT scanner, which is known to have a weaker spectral separation compared to the predominantly used dual-source DECT scanner in this study and the study by Lourenco et al [15, 16, 20]. This made it possibly more challenging to spot subtle hypoenhancement on DECT images. Furthermore, they did not assess reading time, which is particularly significant given their discussion of a potential decrease in overall assessment thoroughness due to an increased number of image reconstructions. They also provided a more complex reading setting for the readers compared to our study. We could show that, independent of the underlying mechanisms, individual readers with increased and decreased reading time can benefit from additional DECT reconstructions and improve their diagnostic accuracy and confidence.

Our study had several limitations. Although it features the largest patient cohort with proven ABI among all radiological studies evaluating DECT imaging performance, the sample size remains moderate due to the rarity of ABI and the study’s single-center design. Only dual-source and split-beam DECT scanners were used in our study, limiting the generalizability of the results for other scanner types, as is demonstrated by the differing results of Xu et al and the concordant results presented by Lourenco et al [15, 16]. A meaningful subgroup analysis of the different scanners included in this study was not performed due to the limited sample size. In our study, the senior and resident radiologist readers are experienced in interpreting DECT images daily. Results may not transfer to radiologists with little or no experience with DECT. Future studies could mitigate these limitations through a multi-centric approach involving larger patient numbers, diverse DECT scanner platforms, and a wider variety of readers. As this is a retrospective study, our standard imaging protocol for suspected mesenteric ischemia, which did not include a true non-contrast scan, was used. Also, other kVp-pairs for the dual-source DECT with better spectral separation (e.g., 80/Sn150 kV) were not investigated in this study. These could improve image quality in iodine maps and VNC images, and lead to even better diagnostic results with DECT images. In future studies, photon-counting detector CT with superior spectral separation could improve image quality and diagnostic accuracy even further. Lastly, readers in our study were not provided with additional coronal or sagittal reconstructions. While this may have lowered overall diagnostic performance, it would have similarly affected conventional and DECT images. However, as previously noted, an increase in the number of reconstructions may compromise the thoroughness of the overall assessment [21]. The lack of coronal and sagittal reconstructions may also account for the low inter-reader agreement observed for SMA/IMA occlusions. In combination with the small caliber of the IMA, especially in NOMI patients, identifying the vessel as patent or occluded can be difficult for inexperienced readers. This underlines why the direct evaluation of the bowel wall perfusion with DECT images helps to diagnose ABI in patients where vessel status is not easily determinable.

In conclusion, our study shows that additional dual-energy CT images, iodine images, and VNC increase diagnostic accuracy and confidence without significantly increasing reading time in patients with suspected ABI.

## Supplementary information

ELECTRONIC SUPPLEMENTARY MATERIAL

## Abbreviations

ABI: Acute bowel ischemia
CI: Confidence intervals
CRP: C-reactive protein
DECT: Dual-energy computed tomography
IMA: Inferior mesenteric artery
IQR: Interquartile range
SMA: Superior mesenteric artery
VMI: Virtual monoenergetic images
VNC: Virtual non-contrast images
WBC: White blood cell count
