# Supplementary material for: Dual-energy CT of acute bowel ischemia—influence on diagnostic accuracy and reader confidence
Source: Eur Radiol. 2024 Nov 27;35(3):1405–14. doi: 10.1007/s00330-024-11217-1 (PMC11836098; doi:10.1007/s00330-024-11217-1)
Supplement: Supplementary file 1 — ELECTRONIC SUPPLEMENTARY MATERIAL [file 330_2024_11217_MOESM1_ESM.pdf]

# Dual-Energy CT of Acute Bowel Ischemia - Influence on diagnostic accuracy and reader confidence
## ELECTRONIC SUPPLEMENTARY MATERIAL

| Body weight (kg) | Contrast volume (mL) |
|---|---|
| 50-55 | 65 |
| 55-60 | 70 |
| 60-65 | 75 |
| 65-70 | 80 |
| 70-75 | 85 |
| 75-80 | 90 |
| 80-85 | 95 |
| 85-90 | 100 |
| 90-95 | 105 |
| 95-100 | 110 |
| >100 | 115 |

Supplementary table 1: Dosage regimen for the application of intravenous contrast medium depending on body weight.
